# Superconductivity in LiGa_2_Ir Heusler type compound with VEC = 16

**DOI:** 10.1038/s41598-021-95944-1

**Published:** 2021-08-13

**Authors:** Karolina Górnicka, Gabriel Kuderowicz, Michał J. Winiarski, Bartłomiej Wiendlocha, Tomasz Klimczuk

**Affiliations:** 1 Faculty of Applied Physics and Mathematics, Gdansk University of Technology, ul. Narutowicza 11/12, 80-233 Gdańsk, Poland; 2 Advanced Materials Centre, Gdansk University of Technology, ul. Narutowicza 11/12, 80-233 Gdańsk, Poland; 3 Faculty of Physics and Applied Computer Science, AGH University of Science and Technology, Aleja Mickiewicza 30, 30-059 Kraków, Poland

**Keywords:** Condensed-matter physics, Theory and computation

## Abstract

Polycrystalline LiGa_2_Ir has been prepared by a solid state reaction method. A Rietveld refinement of powder x-ray diffraction data confirms a previously reported Heusler-type crystal structure (space group *Fm-3m*, No. 225) with lattice parameter *a* = 6.0322(1) Å. The normal and superconducting state properties were studied by magnetic susceptibility, heat capacity, and electrical resistivity techniques. A bulk superconductivity with T_c_ = 2.94 K was confirmed by detailed heat capacity studies. The measurements indicate that LiGa_2_Ir is a weak-coupling type-II superconductor (${\uplambda }$_e–p_ = 0.57, ${\Delta }$C/${\upgamma }$T_c_ = 1.4). Electronic structure, lattice dynamics, and the electron–phonon interaction are studied from first principles calculations. Ir and two Ga atoms equally contribute to the Fermi surface with a minor contribution from Li. The phonon spectrum contains separated high frequency Li modes, which are seen clearly as an Einstein-like contribution in the specific heat. The calculated electron–phonon coupling constant ${\uplambda }$_e–p_ = 0.68 confirms the electron–phonon mechanism for the superconductivity. LiGa_2_Ir and recently reported isoelectronic LiGa_2_Rh are the only two known representatives of the Heusler superconductors with the valence electron count VEC = 16.

## Introduction

With more than a thousand members reported in the literature, the Heusler family remains one of the most interesting and intensively studied intermetallic systems in materials science^1^. Among this class of materials we can find catalysts^2^, ferromagnets^3,4^, thermoelectric^5–7^ and magnetocaloric materials^8^. Unwavering interest in this class of materials is also caused by the various properties and rich physics they offer, such as heavy fermion behavior^3,4,8–10^, shape memory phenomena^11^, magneto-optical^12^ and magneto-structural^13^ effects. Recently, the charge density wave and a quantum critical point were reported in Lu(Pt_1−x_Pd_x_)_2_In solid solution^14^.

What seems to be special for Heusler compounds is that their physical properties can often be predicted just by simply counting the number of valence electrons. This valence electron count (VEC) is frequently used to classify different groups of Heuslers. For example, for VEC = 24 semimetallic behavior is expected^15^ with vanishing net magnetic moment^16–22^. Adding three electrons to the system (VEC = 27) often reveals superconductivity, including the Heusler compounds containing magnetic rare earth metals, i.e. TmPd_2_Sn and YbPd_2_Sn^23^. It is worth noting that most of the known Heusler superconductors, together with T_c_ ~ 5 K record holder YPd_2_Sn, have VEC 27 or 28^24^, the numbers corresponding to 6.5 and 6.75 electrons per atom (el./at.)—exactly at the third maximum of T_c_ proposed for metals by Matthias^25^. The second proposed maximum is between 4 and 6 el./at. and hence the Heusler type superconductors with VEC in this range are of great interest. LiGa_2_Ir and recently reported LiGa_2_Rh^26^ have 4 el./at. and therefore they might belong to the middle maximum in the Heusler family.

The prototype compound MnCu_2_Al was discovered in 1903 by Fritz Heusler and appeared to be a ferromagnet at room temperature. The crystal structure of Cu_2_MnAl was first described more than 3 decades later by James Bradley^27^. The Heusler X_2_YZ compounds form in a cubic space group Fm-3m (s.g. #225) with three occupied Wyckoff positions. The Y and Z atoms are usually the most and the least electronegative metals and they are located in the 4a (0, 0, 0) and 4b (½, ½, ½) sites. The X atoms occupy the 8c position (¼, ¼, ¼) and fill all the tetrahedral holes in the crystal structure. In this special crystallographic site, we can put a few transition metals from group 9, 10, and 11, as well as Li and Mg. However, there are 16 full-Heusler compounds reported with Al, Ga, and In in the 8c site and except UAl_2_Cu and MnGa_2_Co, all of them contain Li. In one of these compounds, LiGa_2_Rh, we recently reported superconductivity^26^. In this paper, we present details of a synthesis process and superconducting properties of the isoelectronic compound—LiGa_2_Ir. This material was obtained by an ordinary solid state reaction without using a Ta tube at rather low synthesis temperature. The observed bulk superconductivity (T_c_ = 2.94 K) was confirmed by the heat capacity, resistivity, and magnetic susceptibility measurements. Theoretical calculations based on Density Functional Theory (DFT) were performed to study its electronic structure, lattice dynamics, and the electron–phonon interaction and allow us to conclude on the electron–phonon mechanism of superconductivity.

## Experimental and computational methods

The polycrystalline LiGa_2_Ir sample was prepared by conventional solid-state reaction. The starting elements were high-purity Li chunks (4N, Alfa Aesar), Ir powder (3N8, Mennica-Metale, Poland), and Ga pieces (3N, Alfa Aesar). First, the precursor of IrGa_2_ taken in a 1:2 molar ratio was placed in an alumina crucible, sealed inside evacuated silica tubes, and annealed at 700 °C overnight. The as-prepared material was thoroughly ground, mixed with Li chunks with 10% excess to compensate for the loss of some Li during the synthesis, and pressed into a pellet using a hydraulic press. Complete sample preparation was performed in an argon-filled glove box system [p(O_2_ ) < 0.5 ppm]. The pellet was then placed in a tantalum crucible in a sealed quartz tube under a partial atmosphere of Ar gas. The tube was heated to 240 °C at a rate of 2.5 °C/h and then heated to 550 °C (10 °C/h), held at that temperature for 6 h, and air quenched to room temperature. The as-prepared material was reground well and once more pressed into a pellet. Finally, the samples were sealed in quartz tubes and annealed at 650 °C for 3 days. The resulting materials formed a soft, brown pellet. The compound, although Li-containing, is stable in air over time and therefore was handled outside of the glovebox for all performed experiments.

Powder x-ray diffraction (pXRD) measurements were performed at room temperature using Cu Kα radiation (λ = 1.5406 Å) on a Bruker D2 Phaser diffractometer with a LynxEye-XE detector. Structure refinement from pXRD data was performed using the Rietveld analysis method using the FullProf package^28^. The magnetization measurements were carried out using a Quantum Design Evercool II Physical Property Measurement System (PPMS) with a Vibrating Sample Magnetometer (VSM) function. The data were collected in the temperature range 1.95–3.2 K under various applied magnetic fields. All thermodynamic and transport measurements were also performed in a PPMS Evercool II system. The heat capacity was measured using the two-τ time-relaxation method in the temperature range 1.85–300 K. Flat, polished, circular samples of around 15 mg were fixed with Apiezon N grease on the α-Al_2_O_3_ measurement platform. The ac electrical resistivity measurements in a temperature range from 1.8 to 300 K were carried out using the standard four-probe method in magnetic fields up to H = 1400 Oe (µ_0_H = 0.14 T). Platinum wires were attached to the surface of the bar-shaped polycrystalline samples using conductive silver epoxy (Epotek H20E). High pressure magnetization measurements were performed using a copper-beryllium bronze, VSM-compatible piston cylinder cell manufactured by HMD. Daphne 7373 oil was used as a pressure transmitting medium. A 15 mg sample was packed together with a small piece of high purity lead wire which was employed as a manometer. For calculating the actual cell pressure the pressure coefficient of the critical temperature for Pb was taken from ref.^29^. Measurements were performed at an applied field of 10 Oe under ZFC conditions.

Ab initio computations were performed for LiGa_2_Ir using density functional theory and Migdal-Eliashberg theory implemented in Quantum Espresso^30–32^. We calculated the electronic structure, phonons and electron–phonon interaction functions. Projector augmented wave pseudopotentials^33,34^ and PBEsol exchange–correlation functional^35^ were chosen. Energy cutoffs of wavefunctions and charge densities were set to 100 Ry and 1000 Ry, respectively. A 12^3^ k-point Monkhorst–Pack mesh was used for self-consistent calculations, whereas the electronic density of states (DOS) and Fermi surface were calculated on 24^3^ grid. Interatomic force constants were calculated on a 6^3^ q-point grid which corresponds to 16 inequivalent q-points in this cubic structure.

## Results and discussion

### Experimental studies

**Figure 1:**
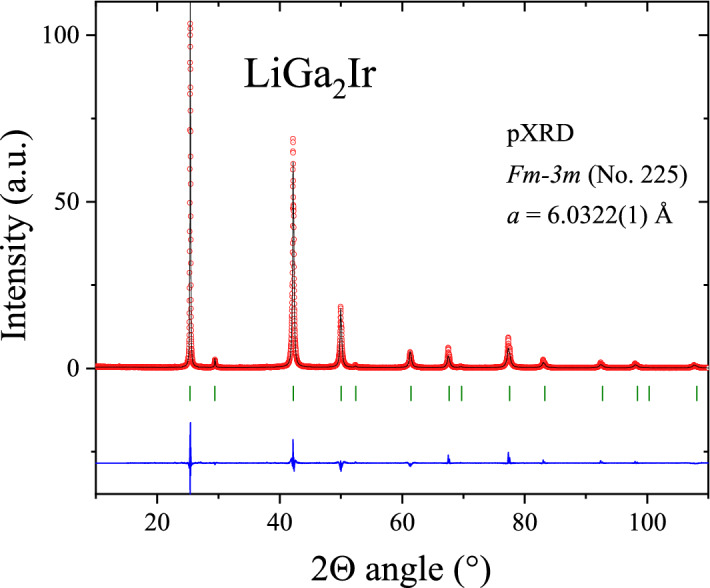
Powder X-ray diffraction pattern of LiGa_2_Ir (red points) together with the Rietveld refinement profile (black solid line). The blue curve is the difference between experimental and model results. The green vertical bars indicate the expected Bragg peak positions (space group *Fm-3m*).

Figure 1 presents the pXRD pattern and results of the Rietveld analysis for the synthesized LiGa_2_Ir. The pXRD analysis indicates an excellent quality of the examined sample and the refinement confirms that the compound crystallizes in the cubic *L2*_*1*_ crystal structure (space group *Fm-3m*, No. 225). A difference plot (between experimental and fitted data) and the Bragg positions are also shown in Fig. 1. The refined lattice parameter *a* = 6.0322(1) Å is in a good agreement with the previously reported for LiGa_2_Ir^36,37^ and slightly larger than refined for LiGa_2_Rh (*a* = 5.9997(8) Å)^26^.

In the analysis, the atomic positions were fixed by symmetry. The refinement of isotropic thermal displacement parameters B_iso_ yielded: 1.7(6) Å^2^, 3.18(7) Å^2^ and 3.16(6) Å^2^ for Li (at the 4*b* site), Ir (4*a*), and Ga (8*c*), respectively. The conventional, background-corrected Rietveld reliability factors for the refinement are R_p_ = 10.9%, R_wp_ = 13.2%, R_exp_ = 7.27%, and χ^2^ = 3.32. A LeBail refinement leads to only slightly lower χ^2^ value (~ 3.3), indicating that the discrepancy between model and observed intensities is dominated by modelling of peak shapes and background and not by structure parameters.

**Figure 2:**
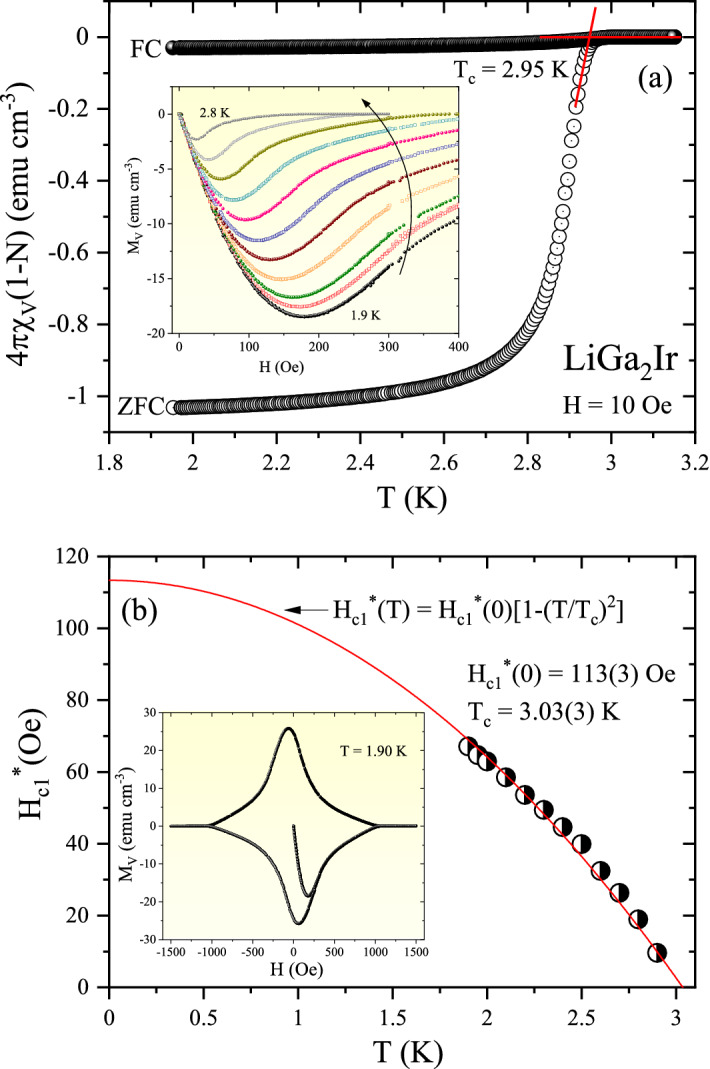
(**a**) Temperature dependences of the zero-field-cooled (ZFC) and field-cooled (FC) volume magnetic susceptibility measured in a magnetic field of 10 Oe. The red straight lines illustrate derivation of the critical temperature. Inset: the field-dependent magnetization curves M_V_(H) for LiGa_2_Ir taken at different temperatures. (**b**) The temperature dependence of the lower critical fields determined from Mv(H). Inset: Magnetization loop at T = 1.90 K.

The superconducting transition of LiGa_2_Ir, was first investigated by the temperature-dependent volume magnetic susceptibility, defined as χ = M/H (M – magnetization, H—applied magnetic field), with zero-field-cooled (ZFC) and field-cooled (FC) measurement modes under H = 10 Oe. As depicted in the main panel of Fig. 2(a), the measured magnetization was multiplied by 4π and corrected for the demagnetization effect − 4πχ_V_ = 1/(1 − N), where the demagnetizing factor N is taken to be 0.58 (estimated from the M_V_(H) fit discussed later). A Meissner transition, corresponding to the onset of superconductivity, appears at T_c_ = 2.95 K, where the superconducting transition temperature (T_c_) was estimated as the point at which the line set by the steepest slope of the magnetization in the ZFC data set intersects with the extrapolation of the normal-state magnetic susceptibility^38^. The shielding volume fraction at 1.95 K is ~ 100%, confirming that the sample is a bulk superconductor. Compared with the ZFC data, the observed FC diamagnetic signal is much weaker, which is likely caused by the porous nature of the polycrystalline sample. In the inset of Fig. 2(a), the low-field regions of the isothermal dc magnetization curves measured at various temperatures ranging from 1.9 K to 2.8 K are presented. For each temperature, the experimental data obtained in low magnetic fields were fitted using a linear relation M_fit_ =  − *a*H, which is suitable for a perfect shielding effect. Assuming that the initial response to the magnetic field is completely diamagnetic, the demagnetization factor N = 0.58 was found. The N value is reasonably consistent with the expected (calculated) N_z_ value derived for a circular cylinder sample with the height to radius ratio of approx. 0.5^39^. At each temperature, the value of the lower critical field ${\text{H}}_{{{\text{c}}1}}^{*}$ is defined as the point of deviation of the data curve from the pure Meissner response. At each T, this point was precisely calculated following the methodology described elsewhere^40^. The estimated ${\text{H}}_{{{\text{c}}1}}^{*}$ values are shown with the corresponding temperatures in the main panel of Fig. 2(b). At T = 1.9 K the ${\text{H}}_{{{\text{c}}1}}^{*}$ is 68 Oe and decreases monotonically with an increase in temperature, to 10 Oe at T = 2.8 K. The data points were analyzed with the equation:

1 $$H_{c1}^{*} \left( T \right) = H_{c1}^{*} \left( 0 \right)\left[ {1 - \left( {\frac{T}{{T_{c} }}} \right)^{2} } \right],$$

where ${\text{H}}_{{{\text{c}}1}}^{*}$(0) is the critical field at 0 K and T_c_ is the superconducting critical temperature. Our experimental data is well described with the above formula and the fit (red solid line) yields ${\text{H}}_{{{\text{c}}1}}^{*}$(0) = 113(3) Oe and T_c_ = 3.03(3) K. Taking into account the demagnetization factor (N = 0.58) derived above, the lower critical field value H_c1_ = ${\text{H}}_{{{\text{c}}1}}^{*}$/(1 − N) = 268 Oe. The obtained value is slightly higher than these reported for the other full-Heuslers compounds^24,26^. The inset in Fig. 2(b) presents the full magnetization loop versus applied magnetic field measured in the superconducting state at T = 1.9 K. It is evident that LiGa_2_Ir exhibits conventional type-II superconductivity.

**Figure 3:**
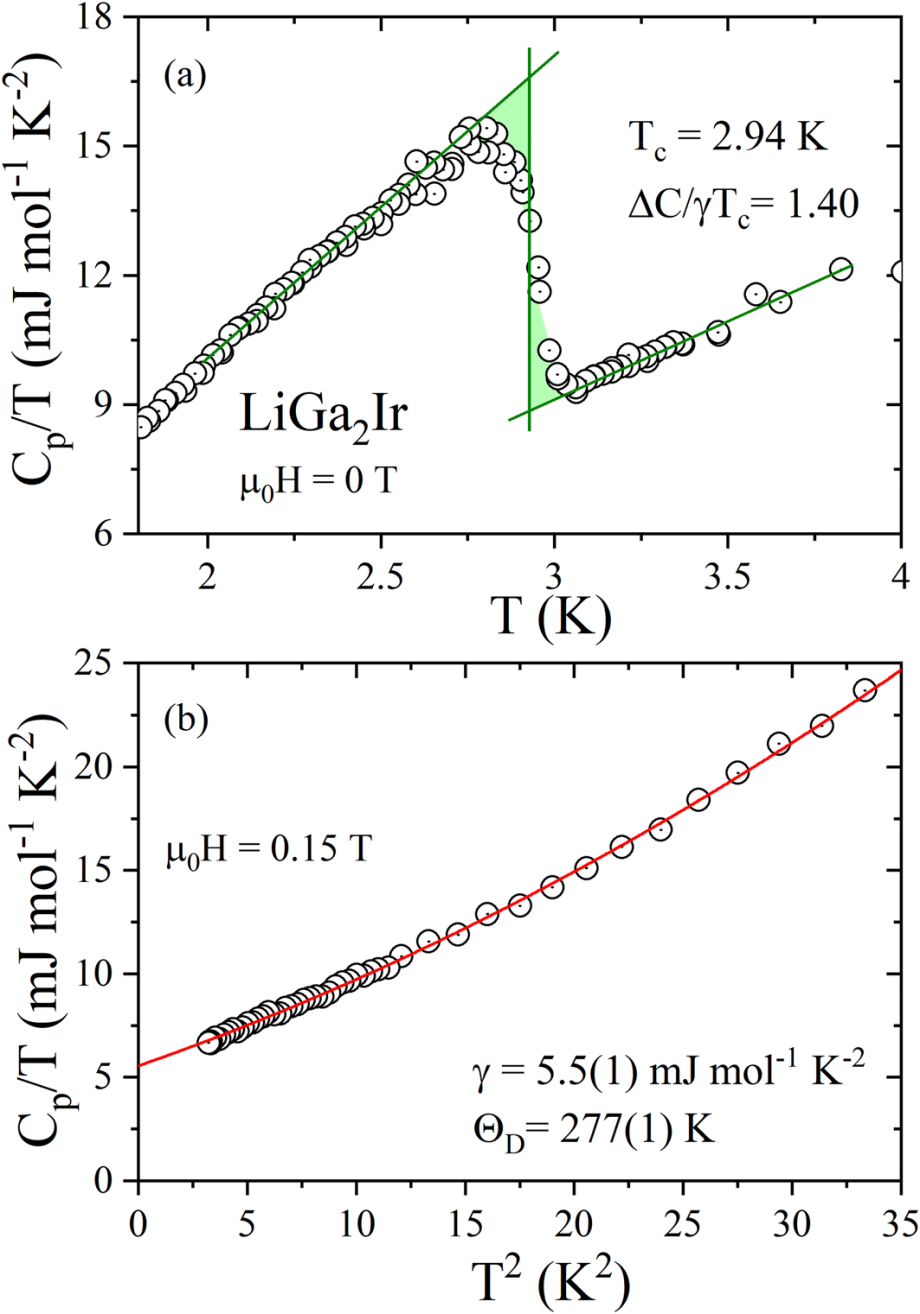
(**a**) Specific heat divided by temperature (C_p_/T ) vs. temperature of LiGa_2_Ir measured in zero magnetic field in the vicinity of the superconducting phase transition. (**b**) C_p_/T vs. T^2^ measured at 0.15 T magnetic field. The red straight line represents the Debye fit discussed in the main text.

The measurement of the heat capacity is reliable evidence of the presence of bulk superconductivity. Figure 3(a) shows in more detail the superconducting transition for LiGa_2_Ir plotted as C_p_/T versus T under µ_0_H = 0 T. The sharp anomaly visible in the specific heat data confirms bulk superconductivity and suggests a good quality of the sample. From the graphical equal-area construction, represented by green solid lines, the T_c_ is estimated to be 2.94 K, which is consistent, with the value determined by magnetic measurements. The specific heat jump at T_c_ is found to be about $\Delta$C/T_c_ = 7.7 mJ mol^−1^ K^−2^. Figure 3(b) shows the heat capacity data plotted as C_p_/T versus T^2^, under µ_0_H = 0.15 T. In the normal state, the raw data can be fitted using the expression C_p_/T = γ + βT^2^ + δT^4^, where the first term is the electronic specific heat coefficient and the second and third terms are attributed to the lattice contributions to the heat capacity (the δT^5^ term in the heat capacity was added after analyzing the computed phonon spectrum, discussed below). The extrapolation, expressed as the red solid line, gives γ = 5.5(1) mJ mol^−1^ K^−2^, β = 0.366(1) mJ mol^−1^ K^−4^ and δ = 0.0052(3) mJ mol^−1^ K^−6^. In a Debye model for the phonon contribution, the β coefficient is related to the Debye temperature Θ_D_ through ${\Theta }_{{\text{D}}} = \left( {\frac{{12\pi^{4} }}{5\beta }nR} \right)^{1/3}$, where R = 8.31 J mol^−1^ K^−1^ and n = 4 for LiGa_2_Ir. The resulting value of Θ_D_ is 277(1) K, which is significantly lower than the Debye temperature for Rh analog LiGa_2_Rh (Θ_D_ = 320 K^26^). Using the previously obtained specific heat jump at T_c_ and the Sommerfeld coefficient (γ = 5.5(1) mJ mol^−1^ K^−2^), the ratio ΔC/γT_c_ = 1.40 can be calculated. The estimated value is close to the BCS value of 1.43, suggesting that LiGa_2_Ir is a weakly coupled superconductor and is close to that of LiGa_2_Rh (ΔC/γT_c_ = 1.48^26^).

The electron–phonon coupling constant λ_e−p_ can be estimated from the McMillan’s equation^41^ by taking the calculated Debye temperature:

2 $$\lambda_{{{\text{e}} - {\text{p}}}} = \frac{{1.04 + \mu^{*} {\text{ln}}\left( {\Theta_{D} /1.45T_{c} } \right)}}{{\left( {1 - 0.62 \mu^{*} } \right){\text{ln}}\left( {\Theta_{D} /1.45T_{c} } \right) - 1.04}}$$

where μ^*^ is the Coulomb pseudopotential factor, usually assumed to be μ^∗^  = 0.13 for conventional intermetallic superconductors^24,42^. Taking T_c_ = 2.94 K and Θ_D_ = 277 K, the calculated λ_e−p_ = 0.57, implying that LiGa_2_Ir can be classified as a weakly coupled BCS superconductor.

**Figure 4:**
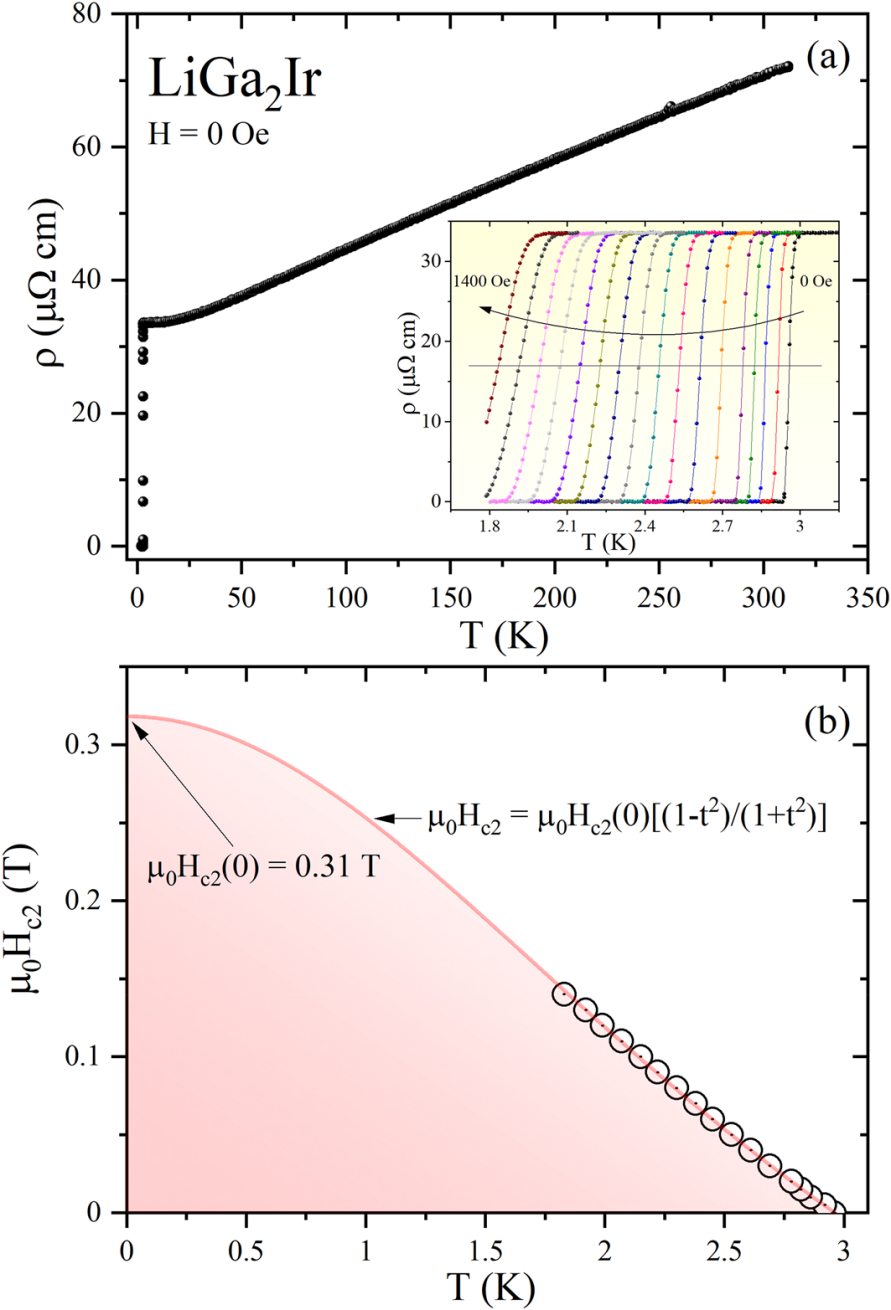
(**a**) The temperature dependent resistivity for LiGa_2_Ir over a wide temperature range measured in zero applied magnetic field. Inset: the low- temperature resistivity data taken in several different magnetic fields. (**b**) The temperature dependence of the upper critical field of LiGa_2_Ir, determined from electrical resistivity measurements.

The temperature-dependent electrical resistivity for LiGa_2_Ir, ρ(T), is depicted in the main panel of Fig. 4(a), in the range of temperature 1.8 − 300 K, without the application of an external magnetic field. In the normal state, the resistivity reveals a metallic behavior (dρ/dT > 0) with rather small residual resistivity ratio (RRR = ρ(300 K)/ρ(5 K) = 2.1). That characteristic can be attributed to the polycrystalline nature of the sample investigated that probably contained many macroscopic defects. The value obtained is comparable to those reported for full-Heusler compounds^24^. The resistivity undergoes a sudden drop at 2.96 K, that perfectly agrees with the T_c_ obtained from magnetic and heat capacity measurements. The inset of Fig. 4(a) emphasizes the low-temperature resistivity under various magnetic fields from 0 to 1400 Oe. As expected, the superconducting transition becomes slightly broader and the T_c_ shifts to a lower temperature as the applied magnetic field is increased. Using the criterion that the point with 50% normal state resistivity (ρ_0_) is the transition temperature, we determined the upper critical field μ_0_H_c2_(T) for LiGa_2_Ir at various temperatures (Fig. 4(b)). The solid line is a fit to the Ginzburg–Landau equation^43^:

3 $$\mu_{0} H_{c2} \left( T \right) = \mu_{0} H_{c2} \left( 0 \right)\frac{{\left( {1 - t^{2} } \right)}}{{\left( {1 + t^{2} } \right)}}$$

where t = T/T_c_ and T_c_ is the transition temperature at zero magnetic field. Equation (3) describes the experimental data well, and yields µ_0_H_c2_(0) = 0.31(1) T. The Pauli limiting field within the BCS theory for a weak electron–phonon coupling^44,45^ gives $H_{c2}^{p} \left( 0 \right) =$ 1.85 T_c_ = 5.4 T, which is eighteen times larger than estimated upper critical field value for LiGa_2_Ir. An identical value of µ_0_H_c2_(0) has been reported for isostructural and isoelectronic LiGa_2_Rh superconductor^26^. Consequently, assuming that the upper critical field is purely orbital, using the GL formula $H_{c2} = \frac{{\Phi_{0} }}{{2\pi \xi_{GL}^{2} }}$ where Ф_0_ = hc/2e is the flux quantum, the superconducting coherence length is calculated to be ξ_GL_ = 322 Å. Similarly, from the relation $H_{c1} = \frac{{\Phi_{0} }}{{4\pi \lambda_{GL}^{2} }}\ln \frac{{\lambda_{GL} }}{{\xi_{GL} }},$ a superconducting penetration depth λ_GL_(0) = 443 Å is found for LiGa_2_Ir. The GL parameter ${\upkappa }_{GL}$ = λ_GL_/ξ_GL_ can then be estimated as ${\upkappa }_{GL}$ = 1.38 > 1/$\sqrt 2$, confirming the type-II nature of the superconductivity. Finally, the thermodynamic critical field can be obtained from κ_GL_, H_c1_ and H_c2_ using the formula $H_{c1} H_{c2} = H_{c}^{2} \ln \kappa_{GL}$. The resulting value of H_c_ is 1633 Oe (µ_0_H_c_ = 0.16 T).

With the Sommerfeld coefficient γ and the electron–phonon coupling parameter λ_e−p_ known, the non-interacting density of states at the Fermi level N(E_F_) can be calculated using the formula:

4 $$N \left( {E_{F} } \right) = \frac{3\gamma }{{\pi^{2} k_{B}^{2} \left( {1 + \lambda_{e - p} } \right)}},$$

where k_B_ is the Boltzmann constant. For LiGa_2_Ir, N(E_F_) is estimated to be 1.49 states eV^−1^ per formula unit (f.u.). Superconducting and normal parameters are gathered in Table 1.

**Table 1:** Experimental superconducting parameters of LiGa_2_T where T = Ir and Rh.

Parameter	Unit	LiGa_2_Ir	LiGa_2_Rh
T_c_	K	2.94	2.4
$\upmu$0H_c1_(0)	mT	26.8	5.9
$\upmu$_0_H_c2_(0)	T	0.31	0.31
$\upmu$_0_H^Pauli^	T	5.4	4.4
ξ_GL_	Å	322	326
λ_GL_	Å	443	2342
$\mathrm{\rm \kappa}$_GL_	–-	1.38	7.2
$\gamma$	mJ mol^−1^ K^−2^	5.5	4.73
ΔC_p_/$\gamma$T_c_	–	1.40	1.48
λ_e−p_	–	0.57	0.52
$\Theta$_D_	K	277	320

**Figure 5:**
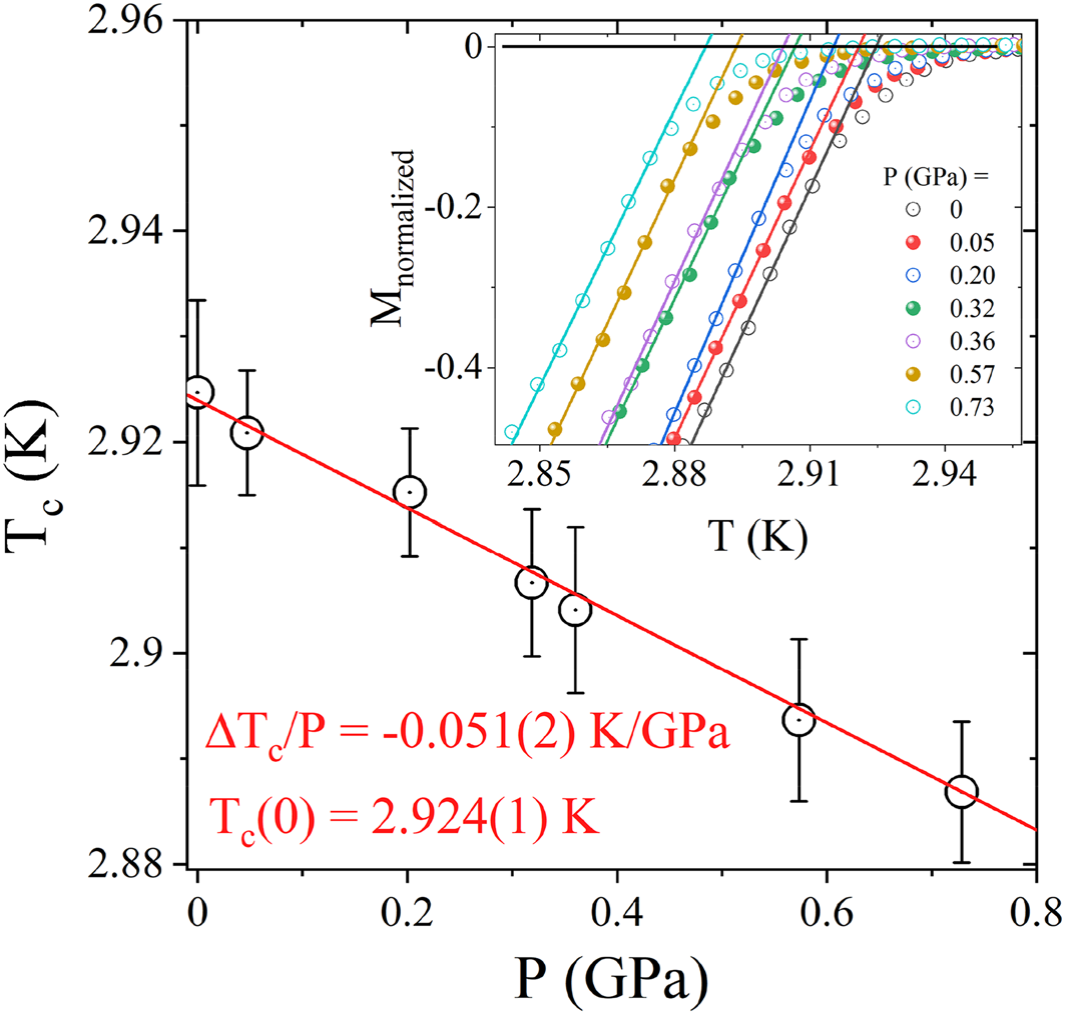
Pressure dependence of the T_c_ for LiGa_2_Ir. T_c_ was taken as the temperature where the extrapolation of the steepest slope of the normalized magnetization versus temperature curves intersects the extrapolation of the normal state magnetization (inset).

Pressure dependence of the T_c_ for LiGa_2_Ir is shown in Fig. 5. LiGa_2_Ir shows an exceptionally low pressure coefficient compared to other Heusler compounds for which high-pressure studies were reported^24,46,47^. It is however worth noting that all of the compounds reported to date are Pd-based systems with a valence electron count of 27 per f.u. As in the case of RPd_2_Z (R = Sc, Y, Tm, Yb, Lu and Z = Sn, Pb) ^47^ and HfPd_2_Al ^24^ the suppression of T_c_ by high pressure likely stems from the stiffening of the lattice, yet in the case of LiGa_2_Ir the effect is much weaker.

### Theoretical calculations

First, the unit cell was relaxed with the Broyden–Fletcher–Goldfarb–Shanno algorithm starting from the experimental lattice constant. Atomic positions were fixed by the symmetry constraints of the full-Heusler structure. The relaxation was repeated including spin–orbit coupling (SOC) because the effect might be important due to the presence of a heavy Ir atom. For calculations with SOC, the scalar-relativistic pseudopotential of Ga and Ir were replaced with the full-relativistic ones. The obtained relaxed lattice constants are in a very good agreement with the experimental one, and SOC was found to have a negligible effect on the lattice constant (Table 2).

**Table 2:** Calculated and experimental lattice constant of LiGa_2_Ir.

	Experiment	w/o SOC	with SOC
a (Å)	6.0322(1)	6.0161	6.0164

**Figure 6:**
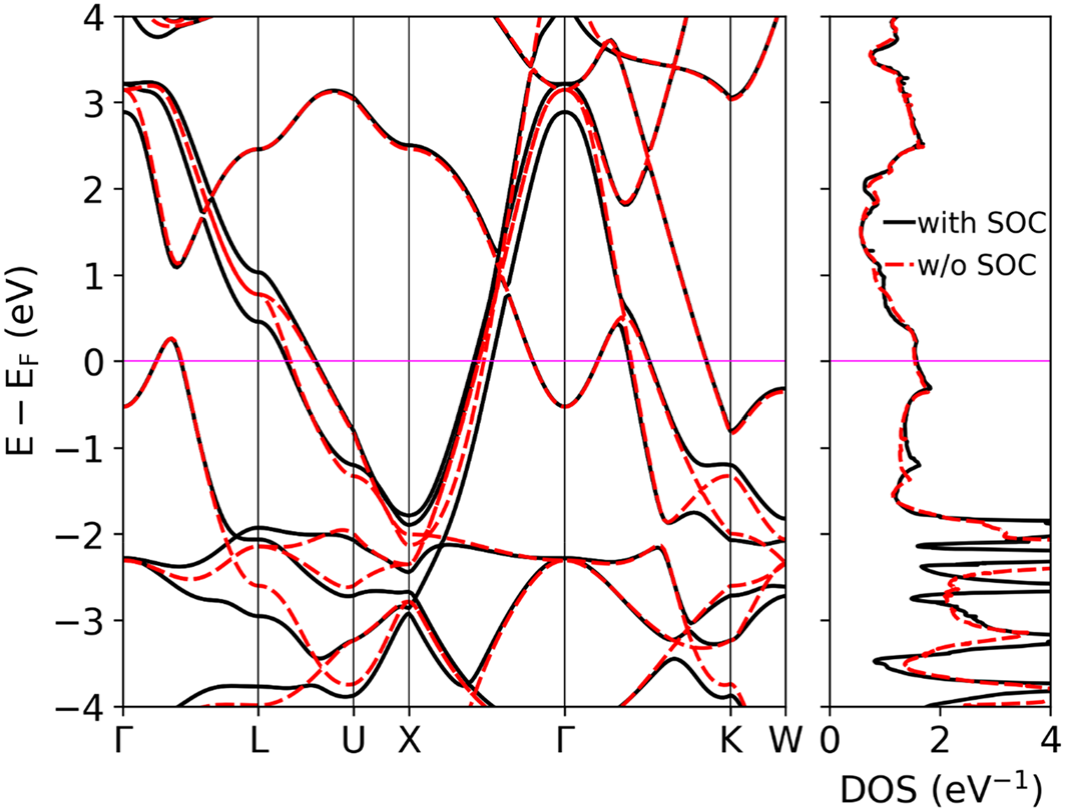
Electronic dispersion relation and total DOS of LiGa_2_Ir calculated with and without SOC.

**Figure 7:**
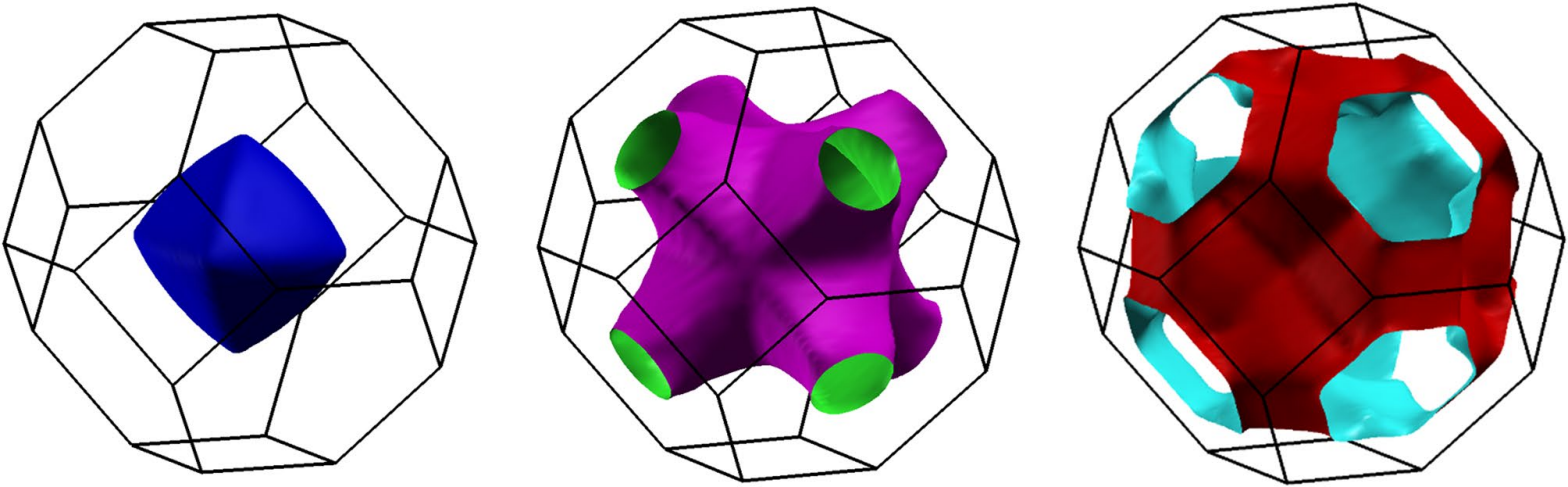
Fermi surface sheets of LiGa_2_Ir.

**Figure 8:**
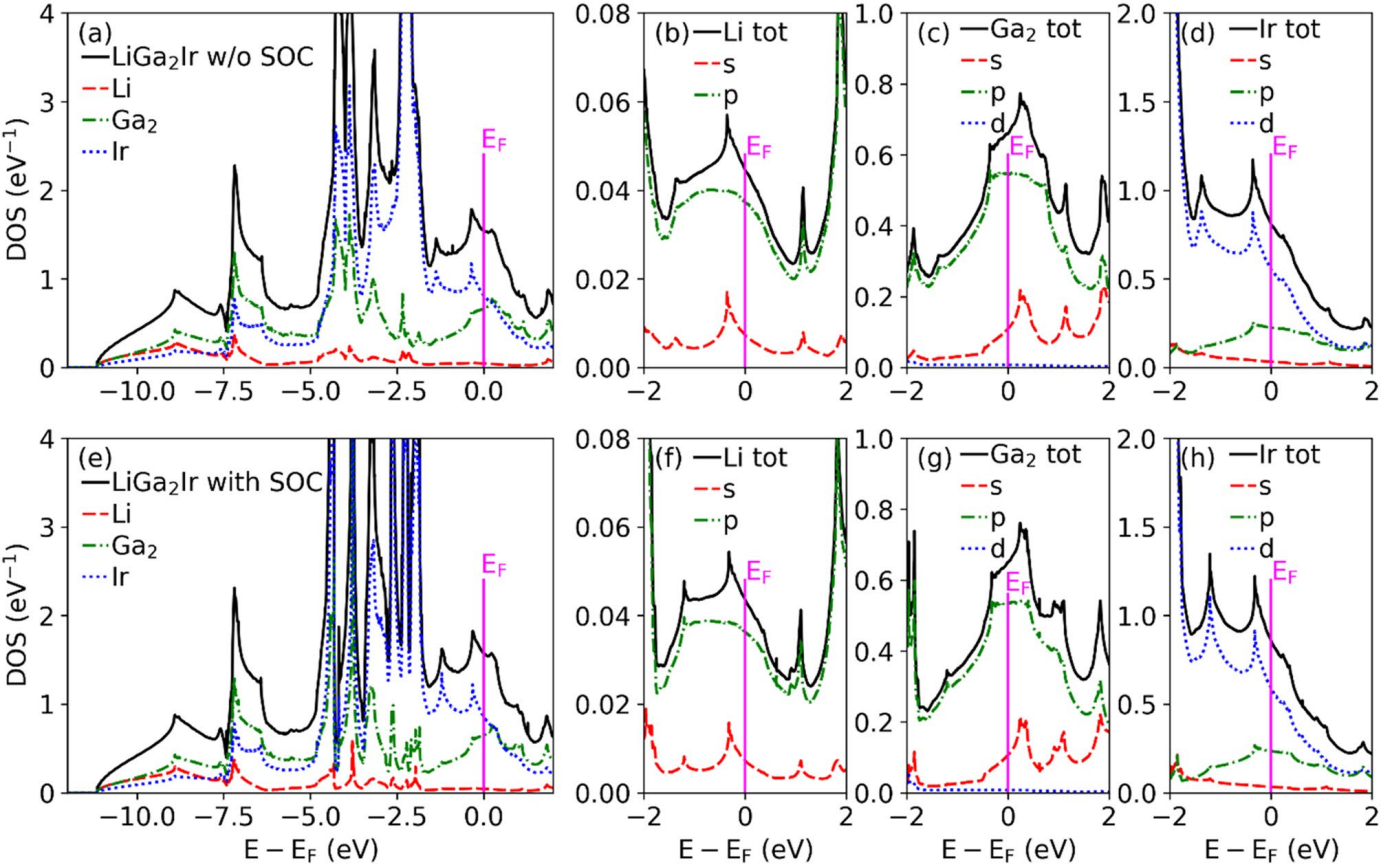
Total and partial DOS of LiGa_2_Ir calculated without SOC (**a**–**d**) and with SOC (**e**–**h**).

Figure 6 shows the electronic dispersion relations and total DOS. Three bands cross the Fermi level forming three Fermi surface sheets visualized using XCrysDen^48^ in Fig. 7. SOC has a small effect on the electronic bands and DOS near the Fermi energy (E_F_), however much stronger SOC effects are seen for electronic states with energies below E_F_, like an anticrossing of bands in the ${\Gamma }$-K direction around −1.5 eV. The total and partial DOS for each atom in LiGa_2_Ir are shown in Fig. 8. States near E_F_ are built mainly from Ga-4*p* and Ir-5*d* orbitals, whereas the contribution to the DOS(E_F_) from Li is negligible. Interestingly, the Fermi level is located in the local minimum of the DOS(E), formed from a superposition of a decreasing DOS of Ir and increasing DOS of Ga. From the calculated DOS(E_F_) values, slightly increased in the relativistic case (see Table 3) , the bandstructure value of the Sommerfeld electronic specific heat coefficient is calculated, $\gamma_{band} = \frac{{\pi^{2} }}{3}k_{B}^{2} DOS\left( {E_{F} } \right)$, and used to estimate the electron–phonon coupling parameter as $\gamma_{expt} = \gamma_{band} \left( {1 + \lambda_{\gamma } } \right)$. This results in $\lambda_{\gamma } = 0.48$, a slight underestimate when comparing to $\lambda_{{{\text{e}} - {\text{p}}}} = 0.57$ obtained from T_c_ using McMillan’s formula.

**Table 3:** Calculated DOS(E_F_), γ_band_ and λ_γ_ of LiGa_2_Ir compared with experimental results obtained from electronic heat and McMillan formula.

	p = 0 GPa		p = 1 GPa
	w/o SOC	with SOC	with SOC
DOS(E_F_) (eV^−1^)	1.545	1.574	1.558
γ_band_ (mJ mol^−1^ K^−2^)	3.64	3.71	3.67
γ_expt_ (mJ mol^−1^ K^−2^)	5.5(1)		–
λ_γ_	0.51	0.48	0.50
λ_expt_	0.57		–

**Figure 9:**
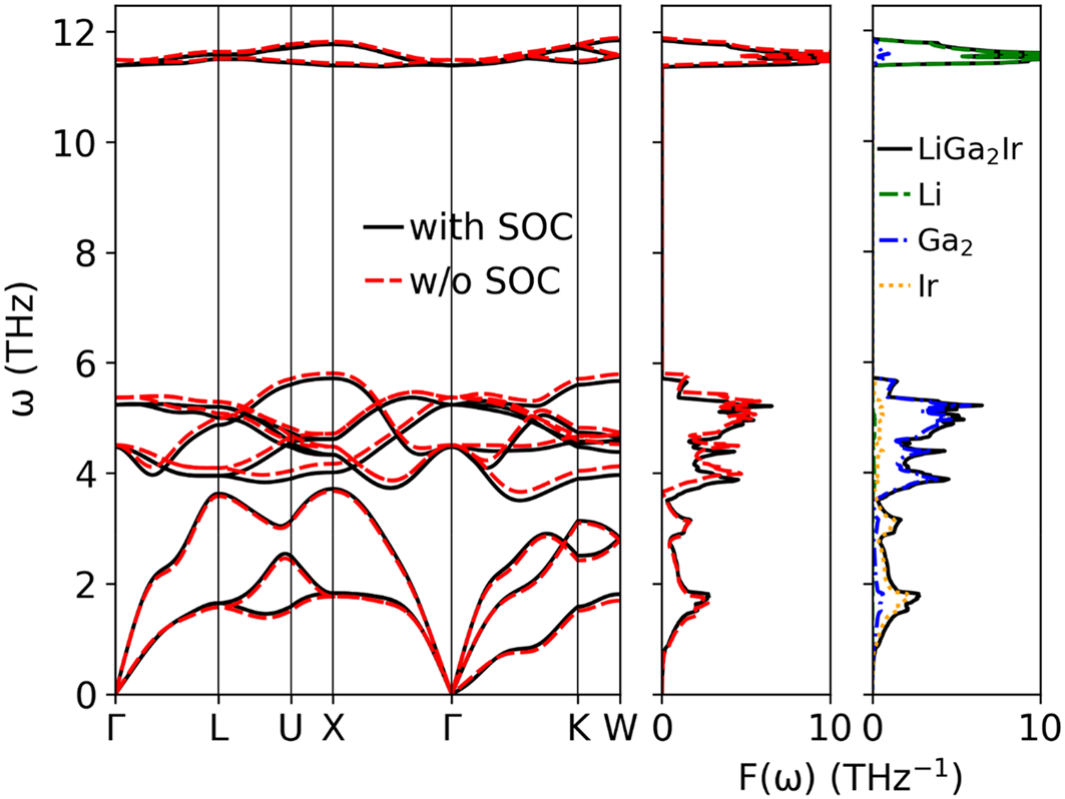
Phonon dispersion relation and density of states of LiGa_2_Ir calculated with and without SOC.

Phonon dispersion relations ${\upomega }$(**q**) and phonon density of states F(${\upomega }$) of LiGa_2_Ir with atomic contributions are shown in Fig. 9. Modes associated with different atoms are well separated because of large differences in mass (M_Li_ = 6.94u, M_Ga_ = 69.72u, M_Ir_ = 192.22u). Three optic modes of Li form an Einstein-like peak in F(${\upomega }$) around 11 THz, a much higher frequency than the Ga, and Ir-dominated parts of the phonon spectrum. The acoustic part is mostly contributed by the heaviest Ir vibrations. Although SOC had a small effect on the electronic DOS(E_F_), it affected phonons, slightly pushing the Ir branches towards higher frequencies and visibly lowering the Ga and Li frequencies. This is shown in Table 4, where the average phonon frequencies are collected. The global effect of SOC is a small decrease in the average frequency, from 5.77 THz to 5.75 THz.

**Table 4:** Calculated average phonon frequencies, electron–phonon coupling constant and superconducting transition temperature from Allen-Dynes formula.

	p = 0 GPa		p = 1 GPa
	w/o SOC	with SOC	with SOC
$\left\langle \omega \right\rangle$(THz)	5.77	5.74	5.82
$\left\langle {\omega _{{Li}} } \right\rangle$ (THz)	11.11	11.08	11.20
$\left\langle {\omega _{{Ga}} } \right\rangle$ (THz)	4.70	4.58	4.65
$\left\langle {\omega _{{Ir}} } \right\rangle$ (THz)	2.67	2.73	2.78
$\left\langle {\omega _{{\log }}^{{\alpha ^{2} F}} } \right\rangle$ (THz)	2.22	2.34	2.35
$\left\langle {\omega _{{\log }}^{{\alpha ^{2} F}} } \right\rangle$(K)	106.64	112.31	112.69
$\lambda$(K)	0.7010	0.6754	0.6708
$T_{{c,calc}} ({\text{K}})(\mu ^{*} = 0.13)$	2.85	2.69	2.64
$T_{{c,calc}} ({\text{K}})(\mu ^{*} = 0.121)$	3.1053	2.9442	2.8963
$T_{{c,expt}} ({\text{K}})$(K)	2.95		–

Looking again at the phonon dispersion relations, we notice the presence of a small dip in the first acoustic branch in the Γ-K direction. Such an anomaly is frequent in Heusler compounds, and was already reported in HfPd_2_Al^49^, LiGa_2_Rh^50^ or LiPd_2_X (X = Si, Ge, Sn)^51^, where in the last case it evolved into a soft mode with an imaginary frequency for X = Ge and Sn. As our ongoing investigation of the LiPd_2_Ge case showed^51^, this may be related to the anharmonic features of the crystal potential.

The theoretical phonon spectrum allows us to analyze the lattice specific heat in more detail. The constant volume C_v_ is computed directly from the phonon density of states F(${\upomega }$) as:

5 $$C_{v} = R\mathop \smallint \limits_{0}^{\infty } F\left( \omega \right)\left( {\frac{\hbar \omega }{{k_{B} T}}} \right)^{2} \frac{{exp\left( {\frac{\hbar \omega }{{k_{B} T}}} \right)}}{{\left[ {exp\left( {\frac{\hbar \omega }{{k_{B} T}}} \right) - 1} \right]^{2} }}d\omega .$$

**Figure 10:**
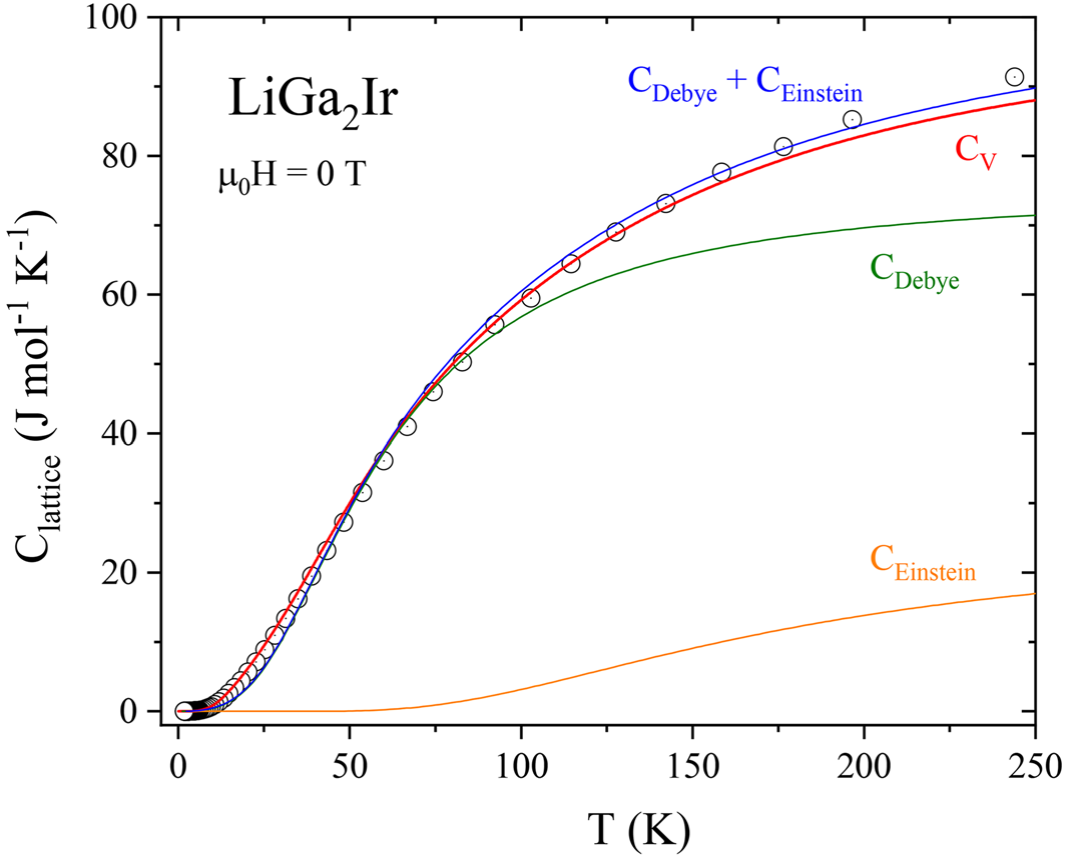
Zero magnetic field, lattice heat capacity data of LiGa_2_Ir versus temperature *T* for 1.85 K < *T* < 250 K. The red line shows constant volume C_v_, computed directly from the phonon density of states F(${\upomega }$). The blue solid line represents the fitted sum of the contributions from the Debye (green) and the Einstein (orange) heat capacity contributions.

The computed curve (red line) is compared to the measured constant pressure C_p_ data (open circles) in Fig. 10, where we notice a good overall agreement.

If one wishes to describe the heat capacity using an approximate model, a combination of Debye and Einstein terms (C_Debye_ + C_Einstein_) should be considered:

6 $$C_{Debye} \left( T \right) = 3n_{D} R\left( {\frac{T}{{\Theta_{D} }}} \right)^{3} \mathop \smallint \limits_{0}^{{\frac{{\Theta_{D} }}{T}}} \frac{{x^{4} exp\left( x \right)}}{{\left[ {exp\left( x \right) - 1} \right]^{2} }}dx,$$

7 $$C_{Einstein} \left( T \right) = n_{E} R\left( {\frac{{\Theta_{E} }}{T}} \right)^{2} exp\left( {\frac{{\Theta_{E} }}{T}} \right)\left[ {exp\left( {\frac{{\Theta_{E} }}{T}} \right) - 1} \right]^{ - 2} ,$$

where *n*_*D*_ and *n*_*E*_ are the number of phonon modes treated as Debye and Einstein type, respectively.

Looking at the phonon DOS in Fig. 9 we see that the three high-frequency Li modes may be described as an Einstein term with the average frequency corresponding to about 540 K, whereas the remaining part, containing nine Ir and Ga modes, could be roughly approximated by the Debye spectrum. Hence, we assume n_D_ = 9 and n_E_ = 3 and we use only two fitting parameters: the Debye and Einstein temperatures. The fit in the temperature range 1.85 K – 200 K gave the values ${\Theta }_{{\text{D}}} =$ 242(1) K and ${\Theta }_{{\text{E}}} =$ 550(10) K. Contributions from each of these terms are shown in Fig. 10 and the combined heat capacity describes the experimental data reasonably well. Deviations are seen in the lower temperature range due to the non-Debye-like phonon spectrum, captured accurately in the direct calculation using Eq. (5). Note that the low-temperature fit, described before, yielded a larger value of Θ_D_ = 277 K as the whole heat capacity was ascribed to the Debye-like phonon spectrum (n = 4 in eq. ${\Theta }_{{\text{D}}} = \left( {\frac{{12\pi^{4} }}{5\beta }nR} \right)^{1/3}$ corresponds to 12 phonon modes, 3 per each of the atom). Since the Einstein term in our case gives no contribution to the specific heat at low temperatures, adopting to the combined model (i.e. changing to n = 3) we get Θ_D_ = 252 K, very close to the value obtained from the fit for the broad temperature range.

**Figure 11:**
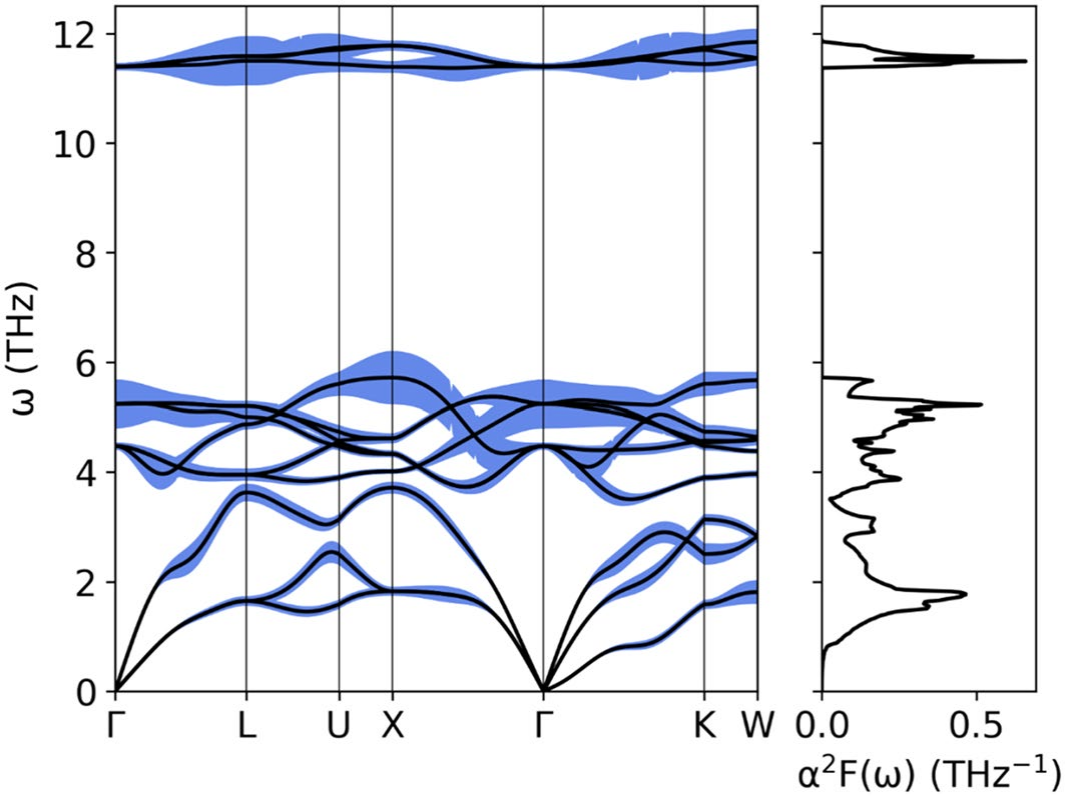
Phonon dispersion relation and Eliashberg function of LiGa_2_Ir calculated with SOC. Blue filling is proportional to phonon linewidth (multiplied 50 times to make it visible).

Moving on to the electron–phonon interactions, the magnitude of the electron–phonon interaction for a given phonon branch is represented in Fig. 11 by the phonon linewidth $\gamma_{{{\varvec{q}}\nu }}$ which is computed from the electron–phonon interaction matrix elements $g_{{{\varvec{q}}\nu }}$ as^52–54^:

8 $$\gamma_{{{\varvec{q}}\nu }} = 2\pi \omega_{{{\varvec{q}}\nu }} \mathop \sum \limits_{ij} \smallint \frac{{d^{3} k}}{{\Omega_{BZ} }}\left| {g_{{{\varvec{q}}\nu }} \left( {{\varvec{k}},i,j} \right)} \right|^{2} \delta \left( {E_{{{\varvec{q}},i}} - E_{F} } \right)\delta \left( {E_{{{\varvec{k}} + {\varvec{q}},j}} - E_{F} } \right),$$

where

9 $$g_{{{\varvec{q}}\nu }} \left( {{\varvec{k}},i,j} \right) = \mathop \sum \limits_{s} \left( {\frac{\hbar }{{2M_{s} \omega_{{{\varvec{q}}\nu }} }}} \right)^{1/2} \langle\psi_{{i,{\varvec{k}}}} {|}\frac{{dV_{scf} }}{{d\hat{u}_{\nu ,s} }} \cdot \hat{e}_{\nu } {|}\psi_{{j,{\varvec{k}} + {\varvec{q}}}}\rangle .$$

In the formulas above, $\omega_{{{\varvec{q}}\nu }}$ is the phonon frequency at the wavevector **q** for the mode ν, $M_{s}$ is mass of atom *s*, $\psi_{{i,{\varvec{k}}}}$ is an electron wavefunction for a given band *i* and wavevector ***k***, $\hat{e}_{\nu }$ is a phonon polarization vector and $\frac{{dV_{scf} }}{{d\hat{u}_{\nu ,s} }}$ is a change of the electronic potential due to a displacement of the atom *s* in the direction *u*.

The strongest electron–phonon interactions, seen as the largest phonon linewidths, are associated with the optic modes of Ga near 5 THz and the Einstein-like Li branch around 11 THz. Next, the electron–phonon interaction function α^2^F(ω) (Eliashberg function) is calculated by summing the contributions from each of the phonon branches, weighted by their inverse frequency:

10 $$\alpha^{2} F\left( \omega \right) = \frac{1}{{2\pi N\left( {E_{F} } \right)}}\mathop \sum \limits_{{{\varvec{q}}\nu }} \delta \left( {\omega - \omega_{{{\varvec{q}}\nu }} } \right)\frac{{\gamma_{{{\varvec{q}}\nu }} }}{{\hbar \omega_{{{\varvec{q}}\nu }} }}.$$

**Figure 12:**
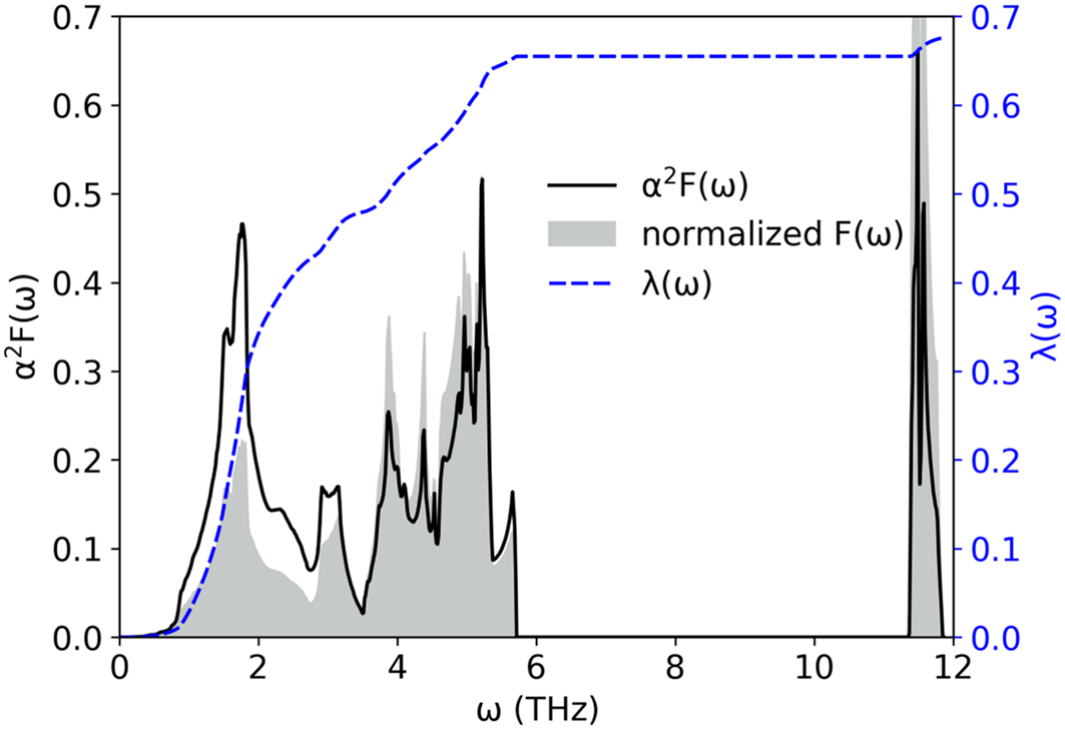
Eliashberg function (solid line) of LiGa_2_Ir and cumulative electron–phonon coupling constant (dashed line) calculated with SOC. Phonon density of states, marked with gray filling, was normalized to have the same integral as Eliashberg function.

α^2^F(ω) is plotted in Fig. 11 and has three peaks associated with enhanced electron–phonon interactions: at 1.78 THz, 5.22 THz, and 11.49 THz . The first peak is associated with acoustic Ir vibrations, having moderate phonon linewidths but low frequencies, effectively increasing the Eliashberg function. The second and third maxima are associated with the above-mentioned Ga and Li branches. Comparing α^2^F(ω) with the phonon DOS function F(ω) (see also Fig. 12) we see, that the Eliashberg function is enhanced over the phonon DOS at lower frequencies, and as a consequence the height of all three α^2^F(ω) maxima become comparable.

The overall electron–phonon coupling parameter ${\uplambda }$ is calculated from the Eliashberg function:

11 $$\lambda = 2\mathop \smallint \limits_{0}^{{{\upomega }_{max} }} \frac{{\alpha^{2} F\left( \omega \right)}}{\omega }d\omega ,$$

and reaches ${\uplambda }$ = 0.68, slightly lowered by the spin–orbit coupling from the scalar-relativistic value of 0.70 (see Table 4). The frequency distribution of ${\uplambda }$

12 $${\uplambda }\left( \omega \right) = 2\mathop \smallint \limits_{0}^{{\upomega }} \frac{{\alpha^{2} F\left( {\omega ^{\prime}} \right)}}{\omega ^{\prime}}d\omega ^{\prime}$$

is plotted in Fig. 12 and the mode contribution from all 12 phonon branches is displayed in Table 5. The acoustic phonons contribute in approximately 72% to the electron–phonon coupling constant, therefore, Ir vibrations are the most important factor in the superconductivity of LiGa_2_Ir. The small and decreasing effect of SOC on the electron–phonon coupling parameter ${\uplambda }$ is related to the small SOC effect on the Fermi surface of the compound and slight increase of the Ir phonon frequencies.

**Table 5:** Electron–phonon coupling constant contributions from the 12 phonon modes.

	λ_tot_	λ_1_	λ_2_	λ_3_	λ_4_	λ_5_	λ_6_	λ_7_	λ_8_	λ_9_	λ_10_	λ_11_	λ_12_
w/o SOC	0.7010	0.2679	0.1588	0.0905	0.0283	0.0291	0.0253	0.0260	0.0235	0.0304	0.0089	0.0058	0.0065
with SOC	0.6754	0.2430	0.1506	0.0873	0.0310	0.0313	0.0271	0.0269	0.0253	0.0324	0.0080	0.0056	0.0067

The superconducting critical temperature T_c_ is calculated using the Allen-Dynes formula^55^:

13 $$T_{c} = \frac{\left<{\omega_{log}^{{\alpha^{2} F}} }\right>}{{1.{20}}}{\text{exp}}\left[ {\frac{{ - 1.{04}\left( {1 + \lambda } \right)}}{{\lambda - \mu^{*} \left( {1 + 0.{62}\lambda } \right)}}} \right],$$

where

14 $$\left< \omega_{log}^{{\alpha^{2} F}} \right> = exp\left( {\mathop \smallint \limits_{0}^{{{\upomega }_{max} }} \alpha^{2} F\left( \omega \right)ln\left( \omega \right)\frac{d\omega }{\omega }/\mathop \smallint \limits_{0}^{{{\upomega }_{max} }} \alpha^{2} F\left( \omega \right)\frac{d\omega }{\omega }} \right).$$

Taking the standard value of the Coulomb screening parameter μ^*^ = 0.13, the calculated value T_c_ = 2.85 K is in a very good agreement with the experimental value of 2.95 K. The experimental T_c_ is exactly reproduced using an only slightly smaller μ^*^ = 0.121. The spin–orbit coupling has a small effect on the calculated critical temperature, slightly decreasing its value to T_c_ = 2.69 K. The agreement between calculations and experiment clearly confirms that superconductivity in LiGa_2_Ir is mediated by phonons.

**Figure 13:**
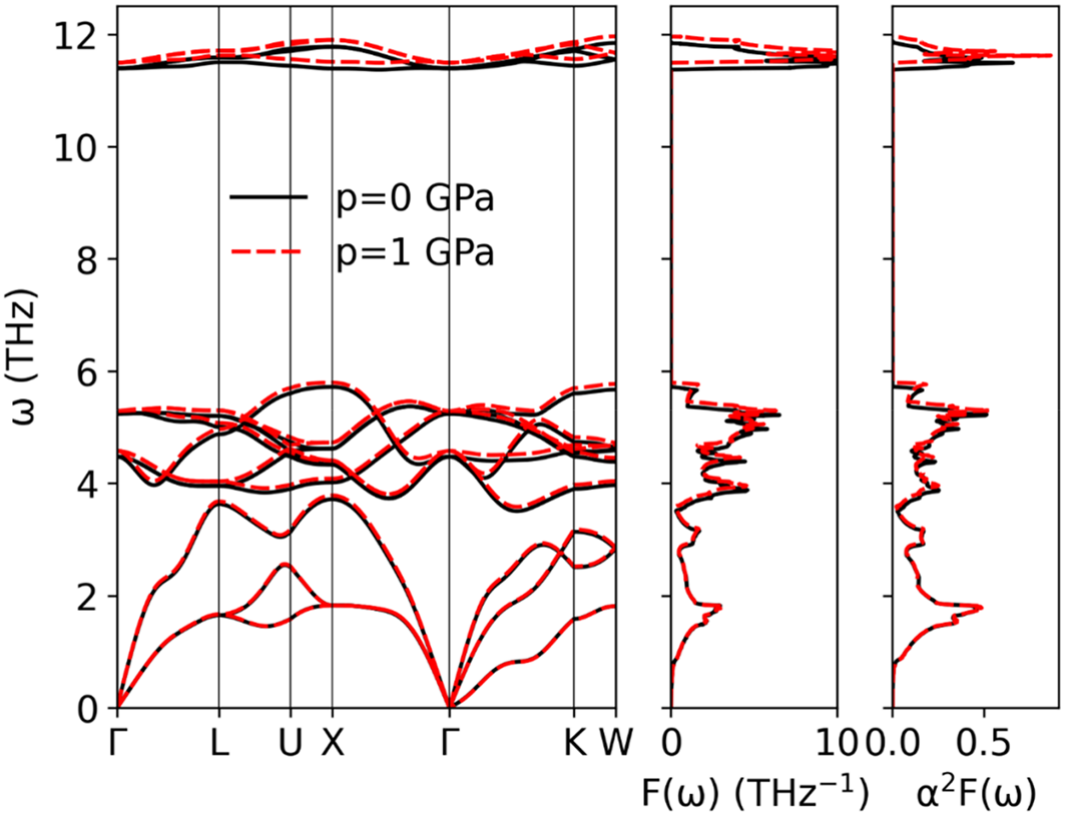
Phonon dispersion relation, phonon density of states and Eliashberg function of LiGa_2_Ir under 1 GPa.

To investigate exceptionally low decrease of T_c_ with pressure we have calculated electronic structure and lattice dynamics under the pressure of 1 GPa. All further calculations were done including SOC. The lattice constant relaxed under 1 GPa is $a = 6.0021$ Å﻿. Electronic structure was almost unchanged, with only slight decrease of DOS(E_F_) by 0.016 eV^−1^ (see Table 3). Moreover, only small changes were induced by the pressure in the phonon dispersion relations and in the electron–phonon coupling. Small effect of the lattice stiffening under the external pressure is observed in phonon dispersion relations shown in Fig. 13, where Ga and Li modes moved towards higher frequencies, but modes of heaviest Ir changed only slightly. The small dip in the acoustic mode at Γ-K was not affected either. Average phonon frequencies, electron–phonon coupling constant and critical temperature are collected in Table 4. Assuming the same value of µ*, which reproduces the experimental T_c_ under ambient pressure, the obtained change of T_c_ is equal −0.048 K/GPa, which is in excellent agreement with the experiment.

To understand why the effect of pressure on T_c_ is so weak in LiGa_2_Ir, comparing to other Heusler compounds, we have to compare the values of characteristic parameters which determine the response of superconductor to external pressure. In our analysis we will compare LiGa_2_Ir to MgPd_2_Sb, for which we have recently found much stronger response to pressure: −0.23 K/GPa^46^. The most important parameter which determines the change of T_c_ with respect to pressure is the variation of the electron–phonon coupling constant ${\uplambda }$, which in the case of LiGa_2_Ir drops from 0.6754 to 0.6708 at p = 1 GPa. On the other hand, in MgPd_2_Sb the change is stronger as ${\uplambda }$ decreases from 0.611 to 0.582. As $\lambda \propto \frac{{\gamma_{q\nu } }}{{\omega_{{{\varvec{q}}\nu }}^{2} }}$ (see Eqs. 6–9) is composed of two factors, the frequency-independent electronic contribution expressed by phonon linewidths $\gamma_{{{\varvec{q}}\nu }}$ and the phonon frequency $\omega_{{{\varvec{q}}\nu }}$, we may analyze what is the origin of such differences. To do so, we calculate the first moment of Eliashberg function ^41^:

15 $$I = \mathop \smallint \limits_{0}^{{\omega_{max} }} \omega \alpha^{2} F\left( \omega \right) d\omega .$$

This quantity is frequency-independent because:

15 $$\begin{aligned} I & = \frac{1}{{2\pi \hbar N\left( {E_{F} } \right)}}\mathop \smallint \limits_{0}^{{\omega_{max} }} d\omega \mathop \sum \limits_{{{\varvec{q}}\nu }} \delta \left( {\omega - \omega_{{{\varvec{q}}\nu }} } \right)\gamma_{{{\varvec{q}}\nu }} \\ & = \frac{1}{{2\pi \hbar N\left( {E_{F} } \right)}}\mathop \smallint \limits_{0}^{{\omega_{max} }} d\omega \mathop \sum \limits_{{{\varvec{q}}\nu }} \delta \left( {\omega - \omega_{{{\varvec{q}}\nu }} } \right)\mathop \sum \limits_{s} \frac{1}{{2M_{s} }} \\ & \quad \times \smallint \frac{{d^{3} k}}{{\Omega_{BZ} }}\left| \langle{\psi_{{i,{\varvec{k}} + {\varvec{q}}}} {|}\frac{{dV_{scf} }}{{d\hat{u}_{\nu s} }} \cdot \hat{e}_{\nu s} {|}\psi_{{j,{\varvec{k}}}} }\rangle \right|^{2} \delta \left( {E_{{{\varvec{k}},i}} - E_{F} } \right)\delta \left( {E_{{{\varvec{k}} + {\varvec{q}},j}} - E_{F} } \right) \\ \end{aligned}$$

At 0 and 1 GPa the first moment of Eliashberg function is equal respectively: 4.144 THz^2^, 4.217 THz^2^ in LiGa_2_Ir and 1.869 THz^2^, 1.890 THz^2^ in MgPd_2_Sb. Thus the changes in *I* are + 1.8% in LiGa_2_Ir and + 1.12% in MgPd_2_Sb. As far as the electronic part of λ is concerned, stronger increase in the electronic contribution in the case of LiGa_2_Ir is found.

Much larger difference is found in the change of the “average square phonon frequency” defined as

17 $$\left\langle {\omega^{2} } \right\rangle = \mathop \smallint \limits_{0}^{{\omega_{max} }} \omega \alpha^{2} F\left( \omega \right)d\omega / \left( {\mathop \smallint \limits_{0}^{{\omega_{max} }} \frac{{\alpha^{2} F\left( \omega \right)}}{\omega }d\omega } \right)$$

With such definition $\lambda = 2I/\left\langle {\omega^{2} } \right\rangle$ and in the case of a weak frequency dependent electron–phonon interaction $\left\langle {\omega^{2} } \right\rangle$ is close to similar quantity determined from the pure phonon DOS^41^. For LiGa_2_Ir we get 12.273 THz^2^ (0 GPa) and 12.574 THz^2^ (1 GPa), i.e. 2.4% increase. On the other hand, for MgPd_2_Sb we have 6.116 THz^2^ (0 GPa) and 6.491 THz^2^ (1 GPa), which is 6.13% increase. When analogical quantities are computed from the pure phonon DOS, the increases are 2.6% for LiGa_2_Ir and 5.5% for MgPd_2_Sb, confirming the trend. Thus, the effect of lattice stiffening takes over the increase in electronic contribution to electron–phonon coupling constant in both materials, explaining the decrease of λ with pressure. The much weaker effect on λ in LiGa_2_Ir is explained by stronger increase in the electronic contribution (parameter *I*) accompanied by the smaller increase in the average square phonon frequency, while compared to MgPd_2_Sb.

Two other global parameters are important in determining the pressure evolution of T_c_. In the McMillan or Allen-Dynes formulas for T_c_ we have $T_{c} \propto \omega_{c}$, with $\omega_{c}$ being the characteristic phonon frequency ($\left< \omega_{log}^{{\alpha^{2} F}} \right>$ in the Allen-Dynes formula and ${\Theta }_{D}$ in McMillan formula). The evolution of the exponential part of T_c_ equation is governed by the evolution of λ, discussed above, thus we should also take a look on how the multiplicator in T_c_ formula is affected by pressure. The more intuitive picture is provided by the McMillan formula, thus calculating the pressure derivative of Debye temperature we get:

18 $$\frac{{\partial \ln {\Theta }_{D} }}{\partial dp} = \frac{1}{B}\frac{{\partial \ln {\Theta }_{D} }}{\partial \ln V} = \frac{{\gamma_{G} }}{B},$$

**Figure 14:**
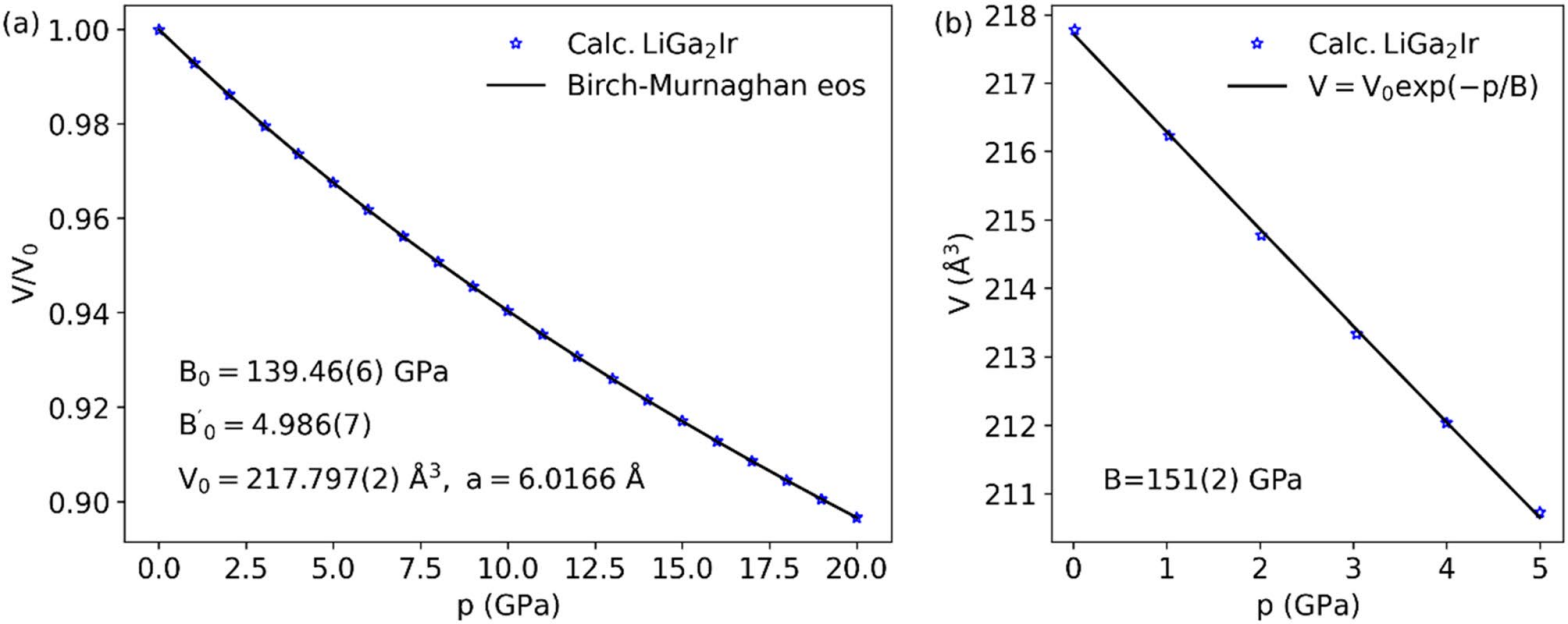
(**a**) Birch-Murnaghan equation of state fitted to calculated cell volumes of LiGa_2_Ir under pressures and (**b**) approximate pressure–volume relation in 0–5 GPa range.

$\gamma_{G}$ is the average Grüneisen parameter:

19 $$\gamma_{G} = - \frac{d\ln \left\langle \omega \right\rangle }{{d\ln V}} \approx \frac{{d\ln {\Theta }_{D} }}{d\ln V},$$

and B is the bulk modulus defined by the approximate pressure–volume relation V = V_0_exp(-p/B) which holds in the small pressure ranges where the variation in bulk modulus with pressure can be neglected. In our case we fitted this relation in the pressure range 0–5 GPa obtaining B = 151(2) GPa (see Fig. 14(b)) for LiGa_2_Ir and 118(2) GPa in MgPd_2_Sb, whereas the average Grüneisen parameter is 1.9 in LiGa_2_Ir compared to 2.7 in MgPd_2_Sb. The larger B, along with the smaller $\gamma_{G}$ additionally lower the pressure dependence of T_c_ in LiGa_2_Ir, compared to MgPd_2_Sb and other Heusler compounds.

To complete the analysis of bulk modulus for LiGa_2_Ir we have fitted p(V) relation using the Birch-Murnaghan equation of state^56^:

20 $$p\left( V \right) = 3B_{0} f\left( {1 + 2f} \right)^{5/2} \left[ {1 + \frac{3}{2}\left( {B_{0}^{^{\prime}} - 4} \right)f} \right],$$

where

21 $$f = \frac{1}{2}\left[ {\left( {\frac{{V_{0} }}{V}} \right)^{2/3} - 1} \right].$$

Pressure volume relation was obtained by relaxing cells up to 20 GPa with a 1 GPa step, whereby the starting lattice constant was the one calculated at p = 0 GPa. Fitted equation of state and fit parameters with standard deviations are shown in Fig. 14(a). Bulk modulus B_0_ equal 139 GPa is quite high and confirms LiGa_2_Ir resistance to pressure. Similarly calculated value for MgPd_2_Sb is 106 GPa, confirming the difference between the two compounds.

## Summary and conclusions

We have made high-quality polycrystalline LiGa_2_Ir using a solid state reaction method. LiGa_2_Ir forms in a full-Heusler crystal structure type with a refined lattice parameter *a* = 6.0322(1) Å, in agreement with that reported by Czybulka, et al. in ref. ^36,37^. The heat capacity, electrical resistivity, and magnetic susceptibility confirm the bulk superconductivity with T_c_ = 2.94 K. Analysis of our data shows that LiGa_2_Ir is a weak-coupling type-II superconductor (${\uplambda }$_e-p_ = 0.57 from the McMillan formula, ${\Delta }$C/$\upgamma$T_c_ = 1.4). Theoretical calculations show that 5*d* states of Ir and 4*p* states of Ga equally contribute to the Fermi surface, which consists of three sheets. Although the compound contains heavy Ir, the spin–orbit coupling does not modify the electronic structure near the Fermi level, with the influence only visible for the deeper-lying electronic states. In the phonon spectrum we may distinguish three groups of modes, lined up according to the atomic mass: high-frequency Einstein-like optic Li vibrations, a medium-frequency group of mostly Ga optic modes and a low-frequency acoustic Ir part. The calculations of the Eliashberg function gave ${\uplambda }$_e-p_ = 0.68 with the dominating contribution from the heaviest iridium. SOC slightly lowers the coupling constant, as the scalar-relativistic value is $\uplambda$_e–p_ = 0.70. The computed superconducting critical temperature agrees very well with the measurements, confirming the phonon mechanism of superconductivity.

Comparing the superconducting properties of LiGa_2_Ir and LiGa_2_Rh (see Table 1) we see that LiGa_2_Ir is another Ir-based superconductor with a T_c_ higher than that observed for the isostructural and isoelectronic compound containing Rh. The other examples are SrRh_2_ vs. SrIr_2_, CaRh_2_ vs. CaIr_2_, IrGe vs. RhGe^57^. This is caused by the larger electron–phonon coupling constants $\uplambda$_e-p_ originating from the larger mass of Ir versus Rh. The heavier atom oscillates with lower frequency, thus the inverse proportionality of enhances λ_e-p_ if in both compounds the frequency-independent phonon linewidths are similar.

The observed weak pressure dependence of T_c_ in LiGa_2_Ir originates from the large bulk modulus, relatively small Grüneisen parameter and compensating increase of the electronic contribution to the electron–phonon coupling constant.

More than 60 years ago, Berndt Matthias proposed that the critical temperature changes with the valence electrons and there are three peaks at VEC ~ 3, 5 and 7 el./at. All known Heusler-type superconductors belong to the third maximum, with two exceptions: LiGa_2_Ir and LiGa_2_Rh^26^ for which VEC = 4 el./at. Superconductivity reported for LiGa_2_Rh^26^ and LiGa_2_Ir (this work) will shed light on the validity of the Matthias T_c_ vs. VEC diagram and can be a stimulus for the future studies and experimental effort to find other Heusler-type superconductors with VEC ~ 20 (5 el./at) for which superconducting transition temperature should be higher.
